# Combination Therapy with TCM Preparation Kumu Injection and Azithromycin against Bacterial Infection and Inflammation: *In Vitro* and *In Vivo*

**DOI:** 10.1155/2022/8533005

**Published:** 2022-03-16

**Authors:** Meiyu Yu, Xuejing Gu, Yanzheng Qu, Fuyou Sun, Yanli Li, Feng Zhao, Hui Xu

**Affiliations:** ^1^Department of Pharmacy, Yantai Muping Hospital of Traditional Chinese Medicine, Yantai 264100, China; ^2^School of Pharmacy, Collaborative Innovation Center of Advanced Drug Delivery System and Biotech Drugs in Universities of Shandong, Key Laboratory of Molecular Pharmacology and Drug Evaluation (Yantai University), Ministry of Education, Yantai University, Yantai 264005, China; ^3^Department of Pharmacy, Binzhou Hospital of Traditional Chinese Medicine, Binzhou 256600, China

## Abstract

**Background:**

Azithromycin (AZM) is one of the most common broad-spectrum antibiotics. However, drug resistance is increasing and combination therapy has attracted great attention. AZM is usually combined with traditional Chinese medicine (TCM) preparations with heat-clearing and detoxifying effects, including Kumu injection (KM) made from *Picrasma quassioides* (D. Don) Benn.

**Purpose:**

The present study aimed to investigate synergistic antimicrobial and anti-inflammatory activities of KM plus AZM with the aim of understanding the mechanism of clinical efficacy of combination regimens.

**Methods:**

Seven common bacterial clinical isolates and LPS-induced RAW 264.7 cells were used for assay of *in vitro* potency. The minimum inhibitory concentration (MIC) was determined for each drug, followed by synergy testing through the checkerboard method and fractional inhibitory concentration index (FICI) for quantifying combined antibacterial effects. The rat model of *Klebsiella pneumoniae*-induced pneumonia was developed and subjected to various drug treatments, namely, AZM, KM, or AZM plus KM, intravenously administered at 75 mg/kg once a day for one week. The combination effects then were evaluated according to pharmacodynamics and pharmacokinetic assessments.

**Results:**

KM-AZM combination synergistically inhibits *in vitro* growth of all the test standard strains except *Pseudomonas aeruginosa* and also the drug-resistant strains of *Staphylococcus aureus, Streptococcus pneumoniae, Shigella dysenteriae, Klebsiella pneumoniae*, and *Escherichia coli*. Despite an additive effect against NO, KM plus AZM at an equal dose could synergistically suppress overrelease of the inflammatory cytokines TNF-*α* and IL-6 by LPS-induced RAW 264.7 cells. The combination significantly inhibited the proliferation of *K. pneumoniae* in the rat lungs, mainly by inactivating MAPKs and NF-*κ*B signaling pathways. KM-AZM combination caused a onefold increase in apparent distribution volume of AZM, along with a significant decrease of AZM level in the livers and heart for pharmacokinetics.

**Conclusion:**

KM-AZM combination displayed synergistic antibacterial and anti-inflammatory effects beneficial to the therapeutic potential against bacterial infection.

## 1. Introduction

Azithromycin (AZM) is a macrolide broad-spectrum antibiotic with ultralong plasma half-life that can inhibit both Gram-positive bacteria and Gram-negative bacteria. Since its discovery, it has been FDA-approved for respiratory tract infections such as pneumonia, genitourinary infections such as chlamydia, and enteric infections such as typhoid, and it has also been extensively studied with malaria [1, 2]. It is widely used clinically for various upper respiratory tract infections caused by *Staphylococcus aureus, Streptococcus pneumoniae*, *Klebsiella pneumoniae,* and other pathogenic bacteria [3]. However, the rate of drug resistance has abruptly increased due to the increase in the types of antibiotic drugs and the expansion of the scope of clinical application. The single drug usually fails to achieve treatment effects and leads to various adverse reactions, which bring great challenges to clinical anti-infective treatment [4, 5].

Traditional Chinese medicine (TCM) preparations containing a variety of biologically active ingredients are widely used in China and could provide new treatment regimens for various intractable diseases [6, 7]. In recent years, the combination of TCM preparations for antibacterial therapy has attracted great interest to deal with drug resistance of antibiotics [8], which mainly involve those heat-clearing and detoxifying TCM compositions [9]. Kumu injection (KM), which is made from the TCM plant *Picrasma quassioides* (D. Don) Benn., has the effects of clearing away lung heat and detoxifying and reducing inflammation and has shown good clinical efficacy for the treatment of various upper respiratory tract infections [10]. However, so far there is no evidence-based support for the therapeutic superiority of KM in combination with AZM.

Our present study just aimed to study the synergistic antimicrobial and anti-inflammatory potency of KM plus AZM from both *in vitro* and *in vivo* investigations. The findings would help to better understand the mechanism of action of AZM-KM combination regimens and lead to a new clinical option for improving AZM related therapy.

## 2. Materials and Methods

### 2.1. Bacterial Strains and Drugs

Reference strains of *Staphylococcus aureus* (CMCC (B) 26003), *Streptococcus pneumoniae* (CMCC (B) 31001), *Hemolytic streptococcus* (CMCC (B) 32210), S*higella dysenteriae* (CMCC (B) 51252), *Klebsiella pneumoniae* (CMCC (B) 46117), *Escherichia coli* (CMCC (B) 44103), and *Pseudomonas aeruginosa* (CMCC (B) 10211) were supplied by China National Institutes for Food and Drug Control (NIFDC, Peking). AZM was purchased from Shandong Luoxin Pharmaceutical Group Co., Ltd. (China). KM was the product of Jiangxi Qingfeng Pharmaceutical Co., Ltd. (Ganzhou, China).

### 2.2. Cell Culture

Mouse monocyte-macrophage RAW 264.7 cells (ATCC: TIB-71, Shanghai Cell Bank, Chinese Academy of Sciences, China) were cultured in RPMI 1640 medium, which was supplemented with 10% heat-inactivated fetal bovine serum, at 37°C in a humidified incubator with 5% CO_2_ and 95% air. The medium was routinely changed every two days. The cells were passaged when they attained approximately 80% confluence.

### 2.3. Animals

Healthy male SD rats (6–8 weeks old, weight 280–320 g) used in this study were supplied by Jinan Pengyue Laboratory Animal Breeding Co., Ltd. (Shandong, China) with the animal certificate of conformity numbered SCXK (Lu) 2014–0007. The feeding environment was specific pathogen-free. The rats were maintained under a 12 h light/dark cycle at 25°C and 50% humidity with free access to sterilized chow diet and water. Animal experimentation was obtained from the Experimental Animal Ethics Committee of Yantai University.

### 2.4. Assay of Bacterial Susceptibility *In Vitro*

The *in vitro* susceptibility test and combination tests were performed as described previously [11]. In brief, the minimum inhibitory concentration (MIC) testing was implemented by using the broth microdilution method. MIC values of AZM and KM were firstly determined by seven bacterial strains. Ultrapure water was used to dissolve and dilute the drugs to give test solutions with the final concentrations ranging from 0.25 to 256 *μ*g/mL for AZM and 62.6 to 4000 *μ*g/mL for KM (total alkaloids). An inoculum of 5 × 10^5^ CFU/mL was obtained by adding 500 *μ*L of 1 × 10^6^ CFU/mL bacterial suspension to the sterile capped test tubes; then, another 500 *μ*L of test solution was pipetted into the tubes. Control was prepared by adding the test bacteria to a tube only containing inert solvent. After overnight incubation at 37°C, the tube containing the lowest drug concentration showing no visible growth was recorded. Then, the MIC values were determined by the broth microdilution method, according to the reference procedure recommended by the Clinical and Laboratory Standards Institute (CLSI) guidelines. Synergy testing of KM combination with AZM was performed by checkerboard method, and interaction was determined according to the fractional inhibitory concentration index (FICI) calculated by the following equation:

(1) $$\begin{array}{c} \text{FICI}=\frac{\;{\text{MIC}}_{A-C}}{{\text{MIC}}_{A}}+\frac{\;{\text{MIC}}_{B-C}}{{\text{MIC}}_{B}}, \end{array}$$

where MIC_*A*_ and MIC_*B*_ were the MIC values for drug *A* or *B* alone and MIC_*A*-*C*_ and MIC_*B*-*C*_ were the MIC values for drug *A* or *B* in combination treatment, respectively [12].

Furthermore, the reference strains on which KM-AZM combination treatment displayed synergic effect were further subjected to *in vitro* induction of AZM resistance by the method of gradually increasing drug concentrations (subinhibitory concentrations) in culture medium [13]. According to CLSI criteria, AZM-resistant strains then were obtained and used to perform bacterial sensitivity tests by the procedure similar to that described above.

### 2.5. MTT Assay

RAW 264.7 cells were seeded in 96-well microplates at a density of 1 × 10^4^ cells/well/200 *μ*L with 5% CO_2_ at 37°C until confluency and then treated with different concentrations of AZM (25, 50, or 100 *μ*g/mL) and/or KM (12.5, 25, 50, 100, or 200 *μ*g/mL) for 24 h, followed by the addition of an MTT solution at a final concentration of 500 *μ*g/mL. Then, the formazan crystals were dissolved in DMSO after incubation at 37°C for another 4 h, and the optical density was measured at 570 nm using a microplate reader. The untreated cells were considered as having 100% cell viability. The results are expressed as a percentage of cell viability when compared with the untreated.

### 2.6. Assay of Inflammatory Cytokines Levels *In Vitro*

Griess method detected the effects of NO released. ELISA method detected the effects of AZM, KM, and KM-AZM on the secretion of cytokines by the cells, respectively. Briefly, RAW 264.7 cells were treated with LPS (1 *μ*g/mL) with or without different concentrations of AZM (25, 50, or 100 *μ*g/mL) and/or KM (12.5, 25, 50, 100, or 200 *μ*g/mL). After 24 h incubation, a total of 100 *μ*L supernatant was removed and mixed with 100 *μ*L of Griess reagent, a mixture of equal volume amounts of 1% sulphanilamide in 5% H_3_PO_4_ and 0.1% naphthyl ethylenediamine dihydrochloride. After shaking the supernatant for 10 min at room temperature, the absorbance at 540 nm was measured, and the concentrations of nitrite were calculated using a prepared standard calibration curve. Meanwhile, a total of 100 *μ*L of the supernatant was removed after 4 h incubation to determine TNF-*α* and IL-6 levels using the commercial ELISA kits according to the manufacturer's recommendations. Then, the *Q* value was further calculated to evaluate the drug combination effect on inflammatory cytokines according to the following equation:

(2) $$\begin{array}{c} Q=\frac{E_{\left(A+B\right)}}{E_{A}+E_{B}-E_{A}\times E_{B}}, \end{array}$$

where *E*_*A*_ and *E*_*B*_ meant the inhibition rate of *A* or *B* used alone and *E*_*(A+B)*_ was that of the *A* and *B* combination at the corresponding concentration [14].

### 2.7. Induction of Pneumonia and Drug Treatment in the Rat Model

*K. pneumoniae* strain was used to establish the experimental pneumonia model by instilling a bacterial dose via the trachea [15, 16]. By comparing cell counts of leukocytes and neutrophils in whole blood (HEMAVET950FS automatic animal blood analyzer) and the gross and microscopic lesions (100% of animals showing lesions with no mortality), the optimum procedure for inducing pneumonia was established as instilling 50 *μ*L of inoculum containing approximately 4 × 10^7^ CFU /mL in the logarithmic growth phase. Then, the model rats were randomly divided into four major groups (*n* = 8 in each group), namely, group Model, AZM, KM, and COM, which was intravenously administered once daily for one week with AZM, KM, AZM plus KM, or equivalent vehicle (physiological saline), respectively, and one dose was 75 mg/kg for each drug. The animals in group NC were used as normal control without any treatment, including bacteria instilling and drug administration.

### 2.8. Biosamples Collection

For the animals in groups AZM and COM, the blood was periodically collected via orbital venous plexus within 24 h after the last dosing, then separated using refrigerated centrifugation (15 000 rpm × 10 min) to afford the plasma, snap-frozen, and stored at −80°C for further analysis. All the animals then were sacrificed, and half rats in each group were randomly taken for lung lavage through the tracheal cannula. Briefly, the airway was washed three times with 1 mL of PBS (pH 7.4) containing 136.89 mM NaCl, 2.67 mM KCl, 8.24 mM Na_2_HPO_4_, and 1.76 mM KH_2_PO_4_; then, the bronchoalveolar lavage fluid (BALF) was collected from each rat, pooled and centrifuged at 4°C (13 000 rpm × 10 min) to obtain supernatant, and stored at −80°C for further analysis. Meanwhile, lungs were removed aseptically from the other half animals in each group, weighed, homogenized in sterile physiological saline, and subjected to further analysis.

### 2.9. Histopathological Examination

After BALF collection, the ligated left lung was fixed with 10% (v/v) neutral formalin for 24 h for histopathological examination [17, 18]. The lungs were placed in a rubber-capped ampulla bottle filled to a 2/3 maximum volume level with 10% (v/v) neutral formalin to enhance fixation. Then, the lung tissues were paraffin-embedded, sectioned at 4 mm and sliced (5 *μ*m), and then stained with hematoxylin & eosin (HE) for light microscope observations. All specimens were analyzed, and the representative images were captured by two pathologists with the blind investigation. Alveolar congestion, infiltration or aggregation of inflammatory cells in airspaces or vessel walls, and the thickness of the alveolar walls were assessed.

### 2.10. Bacterial Colony Counting and Assay of Inflammatory Cytokines

Bacteriological measurement was performed by using serial dilutions of the lung homogenates, which were plated onto Mueller-Hinton agar, and CFU counting was carried out after overnight incubation at 37°C. Both BALF and lung tissue homogenates were subjected to assessment of NO, TNF-*α,* and IL-6 levels according to the above-mentioned methods for *in vitro* assay.

### 2.11. Western Blot Analysis

Total proteins were extracted from lung tissues (20 mg) using the RIPA lysis buffer following the manufacturer's instructions. The protein concentrations were measured at 562 nm using the bicinchoninic acid (BCA) protein assay kit (Solarbio, Beijing, China). Equal amounts of protein were loaded onto sodium dodecyl sulfate-polyacrylamide gel electrophoresis (SDS-PAGE) and transferred to a polyvinylidene difluoride (PVDF) membrane (Millipore, Bedford, MA, USA). The membrane was blocked in Tris-buffered saline-Tween (TBST, pH 7.4) containing 5% skim milk at room temperature for 2 h and then incubated with the corresponding antibodies for ERK 1/2 (1 : 1000), p-ERK 1/2 (1 : 1000), p38 (1 : 1000), p-p38 (1 : 1000), JNK (1 : 1000), p-JNK (1 : 10000), I*κ*B *α* (1 : 10000), p-I*κ*B *α* (1 : 10000), p65 (1 : 1000), p-p65 (1 : 2000), and GAPDH (1 : 10000), respectively. GAPDH was used as an internal reference to analyze the relative expression of the protein. After the membranes were washed with TBST (pH 7.4), they were incubated with HRP conjugated goat anti-rabbit IgG (H + L) (1 : 10000) for 1 h at room temperature. The PVDF membranes were detected using enhanced chemiluminescence reagent (ECL) and exposed to Kodak photographic films; target bands are grayscaled by Image J.

### 2.12. Quantification of AZM and Pharmacokinetic Analysis

The AZM concentration in plasma or tissue was determined by a UPLC-MS/MS method previously reported after sample preparation via acetonitrile protein-precipitation [19]. Briefly, an AB Sciex Triple Quad™ 4500 system (Applied Biosystems Inc., Foster City, CA) was connected to Shimadzu LC-30AD (Shimadzu Corporation, Kyoto, Japan) via electrospray ionization (ESI) interface. The mass spectrometer was operated in positive mode by using multiple reaction monitoring (MRM) of the transitions *m/z* 749.4/591.5 (AZM) and 544.2/396.8 (IS, doxorubicin). The method was fully validated according to current FDA guidelines, and the Analyst 1.5.2 Software (Applied Biosystems Inc.) was employed to process the data. The plasma concentration-time data were analyzed with noncompartmental model by DAS 2.1 software (Mathematical Pharmacology Professional Committee of China, Shanghai) to obtain the pharmacokinetic parameters, including area under the plasma concentration versus time curve (AUC), half-life (t1/2), elimination rate constant (k), and mean residence time (MRT).

### 2.13. Statistical Analysis

Statistical analysis was performed by using the statistical software package SPSS 20.0 (International Business Machines Corporation, New York, USA). All data are expressed as the means ± standard deviation (SD) and the differences between the groups were analyzed using one-way analysis of variance (ANOVA). The significance of the results is depicted in all graphs and tables as a *P* value of <0.05 was considered to indicate a statistically significant difference.

## 3. Results

### 3.1. *In Vitro* Antibacterial Effect

The *in vitro* antibacterial effect of the KM-AZM combination was investigated for various bacteria strains, and the results such as MIC and FICI values are listed in Table 1. According to the FICI values, the combination medication showed no effect on the standard strain of *P. aeruginosa* but showed an addition effect against *H. streptococcus*. For the other five standard strains of *S. aureus, S. pneumoniae, S. dysenteriae, K. pneumoniae*, and *E. coli*, a synergistic effect of the KM-AZM combination could be observed with an FICI value of not more than 0.5.

Then, the five strains were induced by AZM treatment to obtain AZM-resistant strains for further evaluation. As shown in Table 1, the MIC values of AZM to these resistant strains were all not less than 256 mg/L, indicating that all the five strains were indeed highly resistant to AZM according to the CLSI standard [20]. Fortunately, it could be observed that KM and AZM combination showed a synergistic inhibitory effect on these AZM-resistant strains; the most potent inhibition was found on the resistant strain of *K. pneumonia* with the lowest FICI of 0.37.

### 3.2. *In Vitro* Anti-Inflammatory Effect

**Figure 1:**
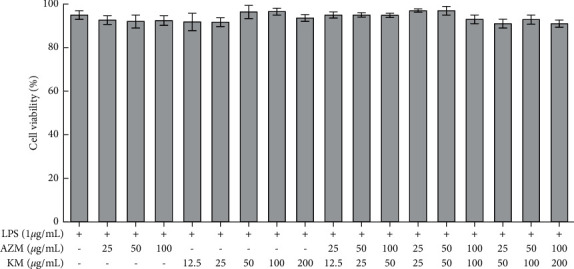
Effect of various treatments on RAW264.7 cell viability *in vitro*.

The *in vitro* anti-inflammatory effect was investigated by using an LPS-induced RAW264.7 cell model according to the assessment of release of several major cytokines such as NO, TNF-*α,* and IL-6. As illustrated in Figure 1, all the treatments showed a cell survival rate detected by the MTT method greater than 90%, and there was no obvious difference compared with the group only treated with LPS at the same concentration (*P* > 0.05). It thus demonstrated that neither the single drug group nor the combination drug group would affect the normal proliferation of RAW264.7 cells.

**Figure 2:**
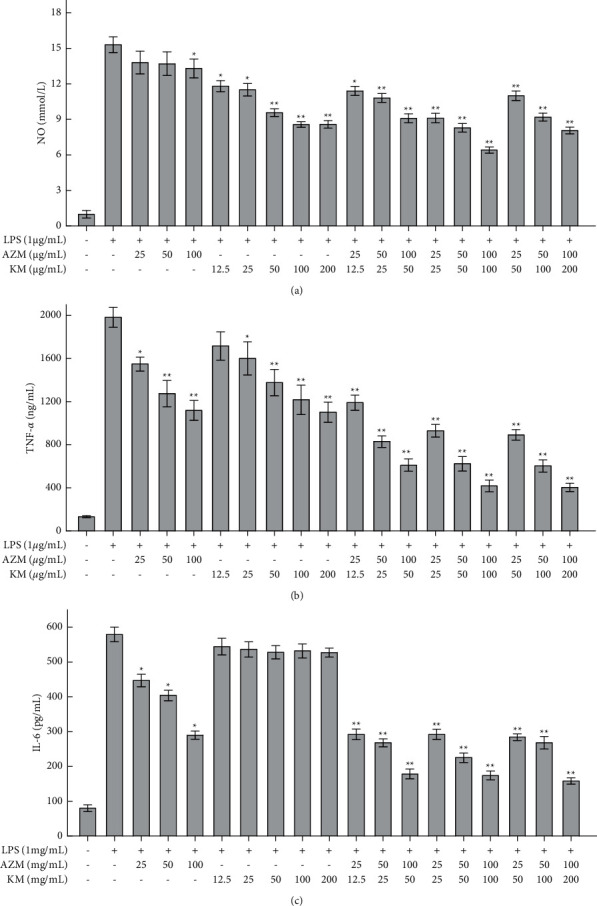
Effect of various treatments on LPS-induced RAW 264.7 cells releasing inflammatory cytokines such as NO (a), TNF-*α* (b), and IL-6 (c), ^*∗*^*P* < 0.05*vs* LPS-treated group; ^*∗∗*^*P* < 0.01*vs* LPS-treated group.

However, the combination of AZM and KM could suppress overrelease of the inflammatory factors NO, TNF-*α,* and IL-6 induced by LPS in RAW264.7 cells. More to the point, the inhibitory effect changed with the dose ratio of the two drugs, and the KM-AZM combination showed more potent inhibition than the single drug KM or AZM did (Figures 2(a)–2(c)). It could be found that AZM plus KM at a mass ratio of 1 : 1 exerted the most potent inhibitory effect against LPS-induced overrelease of these inflammatory factors.

The *Q* value was further calculated to evaluate the anti-inflammatory effect of the KM-AZM combination at various mass ratios. As shown in Table 2, the KM-AZM combination displayed an additive effect on NO with the Q value of 0.87∼0.97 while a synergistic effect on both TNF-*α* and IL-6 with the Q values of 1.16∼1.39 and 1.17∼1.42, respectively.

### 3.3. *In Vivo* Effect on Lung Colony Counts

**Figure 3:**
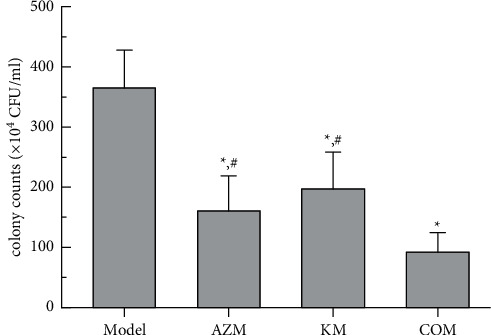
Bacterial clearance rate of lung tissue under different treatments. ^*∗*^*P*  <  0.05*vs* Model group; ^#^*P* < 0.05*vs* COM group.

As to the effect of antibacterial activity *in vivo* on a rat pneumonia model, compared with the model group, the number of colonies in the single drug group and the combination group was significantly reduced, but no significant difference was found among the two single drug groups (*P* > 0.05). More importantly, in contrast to the single drug group, the number of colonies in the combination group was significantly lower (*P* < 0.05, Figure 3). The number of homogenized colonies in the lung tissue measured by the combination group was reduced by 85% compared to the model group and 47% compared with the single drug group. The result shows the combination group had the strongest antibacterial activity *in vivo*.

### 3.4. *In Vivo* Anti-Inflammatory Therapeutic Efficacy

**Figure 4:**
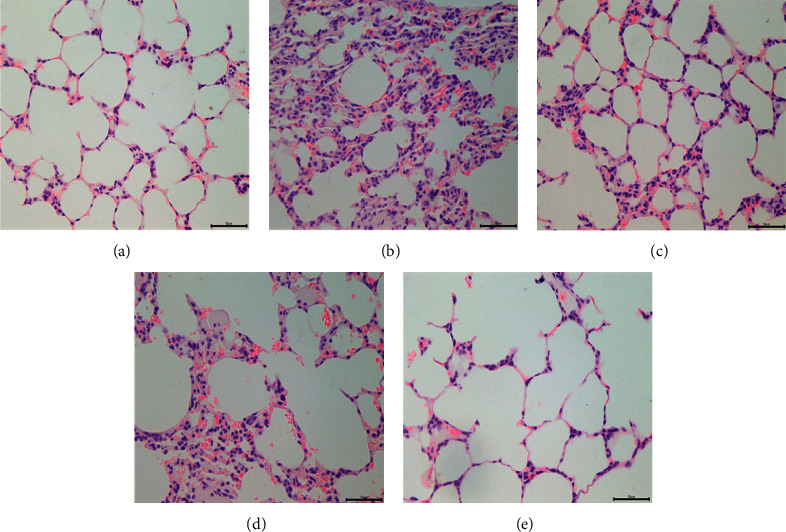
The histopathological changes of rat lung tissue were examined at 24 h after treatment (HE, ×400). NC group (a); model group (b); AZM group (c); KM group (d); COM group (e).

As shown in Figure 4, considerable differences among various treatments were further observed by histological examination of lung tissues. The alveolar tissue was normal in the normal group, the alveolar septum was not thickened, and there were no abnormal infiltration and proliferation (Figure 4(a)). The mucosal epithelial cells were arranged neatly and orderly, and no obvious inflammatory cell infiltration was observed inside and outside the official cavity. After LPS stimulated infection, in the model group, the alveolar interval was significantly widened, there was infiltration in a large number of inflammatory cells, blood vessels were dilated and congested, and the alveolar cavity was expanded (Figure 4(b)). Part of the alveolar expansion and fusion in the AZM group showed a small amount of inflammatory cell infiltration, and the alveolar interval was significantly reduced compared with the model group (Figure 4(c)). Part of the alveolar structure of the KM group disappeared with a small amount of inflammatory cell infiltration and hyperemia (Figure 4(d)). The alveolar interval was reduced compared with the model group, but the treatment effect was not as good as that of AZM. A small amount of inflammatory exudate was seen in the alveolar of the combination drug group; the inflammatory cell infiltration and alveolar interval were significantly reduced compared with the model group (Figure 4(e)).

**Figure 5:**
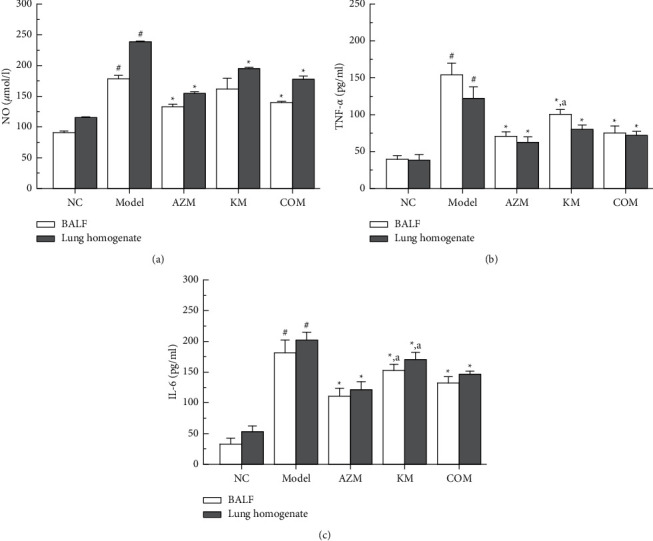
The levels of inflammatory cytokines in BALF and lung homogenate such as NO (a), TNF-*α* (b), and IL-6 (c), ^*∗*^*P* < 0.05*vs* model group; ^#^*P* < 0.05*vs* NC group, and ^a^*P* < 0.05*vs* COM group.

Compared to the NC group, the rats in the model group showed marked increases in inflammatory cytokines including NO, TNF-*α,* and IL-6 (*P* < 0.05, Figures 5(a)–5(c)). After different treatments for *K. pneumoniae* infections, there was a significant decrease in the levels of NO, TNF-*α,* and IL-6 in BALF and lung tissue homogenate. Compared with the KM group, the combined administration can significantly reduce the level of TNF-*α* and IL-6 in BALF and the level of TNF-*α* in lung homogenate. The combination drug showed a stronger anti-inflammatory effect than the single drug.

### 3.5. Regulating Effect on MAPKs and NF-kB Pathways

**Figure 6:**
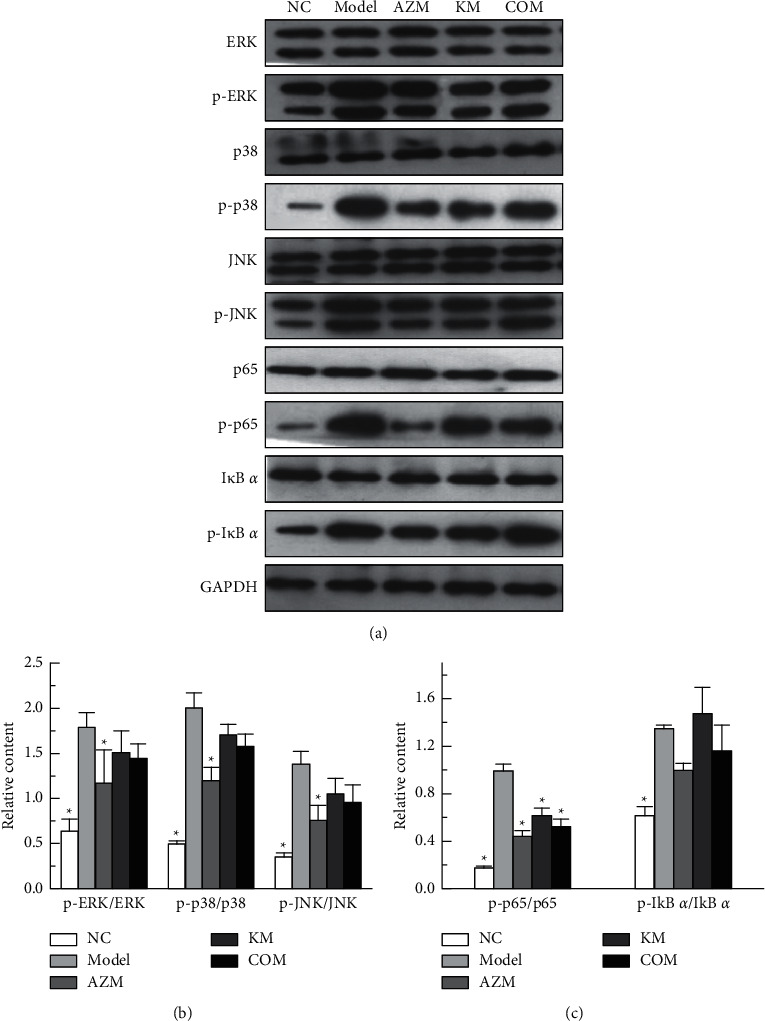
(a) Protein expression. (b) Effect of AZM and KM on the phosphorylation of ERK, p38, and JNK. (c) Effect of AZM and KM on the phosphorylation of p65 and I*κ*B *α* proteins. ^*∗*^*P* < 0.05*vs* model group.

*K. pneumoniae* obviously induced the phosphorylation of ERK, JNK, I*κ*B *α*, p38, and p65 proteins, while all treatment reduced the phosphorylation of ERK, JNK, I*κ*B *α*, P38, and p65 to some extent (Figures 6(a)–6(c)). The activation of these key proteins means that the signaling pathway is activated. In more detail, compared to the model group, the AZM treatment group effectively suppressed the phosphorylation of JNK, p38, and p65 proteins, whereas the inhibition of the phosphorylation of I*κ*B *α* and ERK proteins was not prominent. The KM and COM group treatment had no significant effect on the protein expression of p-ERK, p-JNK, p-p38, and I*κ*B *α*, while it had a significant effect on the protein expression of P65 proteins. Therefore, our data have shown that the difference between the treatment groups means that the combination of AZM and KM attenuated inflammation by inactivating MAPKs and NF-*κ*B signaling pathways and thus attenuated inflammation caused by *K. pneumoniae* infection.

### 3.6. Pharmacokinetics and Biodistribution Profile in Model Rats

**Figure 7:**
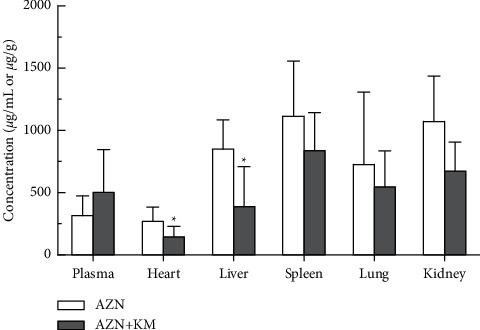
Distribution of AZM in various tissues after administration. AZM in plasma (*μ*g/ml) or tissue (*μ*g/g). ^*∗*^*P* < 0.05*vs* AZM group.

The major pharmacokinetic parameters of the AZM calculated by noncompartmental method are summarized in Table 3. Multiple-dose coadministration of KM changed the pharmacokinetic profile of AZM in rats. However, in contrast to the AZM (4.93 h) group alone, the MRT of the COM group (8.38 h) was significantly different. Meanwhile, the Vd of the COM group was significantly higher than AZM alone; the values are 92.83 L·kg^−1^h and 150.95 L·kg^−1^, respectively. The results of AZM concentration measurement in each tissue after administration showed that 24 h after the last administration, compared with the AZM group, the concentration of AZM in the COM group decreased significantly in the liver and heart (*P* < 0.05, Figure 7), and there was no significant difference in plasma and other tissues. The combination of KM and AZM may accelerate the metabolism of AZM in the heart and liver of pneumonia rats, thereby reducing the accumulation of drugs in the heart and liver, which is beneficial to alleviate the adverse reactions of AZM.

## 4. Discussion

AZM is the most widely used macrolide antibiotic. Unfortunately, with the widespread use of antibiotics, the side effects and number of drug-resistant strains are increasing, which have seriously compromised clinical efficacy [21–23]. Due to the multitarget and multipathway antibacterial action mechanism of traditional Chinese medicine preparations compared to antibiotics, antibiotics combined with traditional Chinese medicine preparations to improve drug resistance have become very common clinically [24, 25]. The combination of Chinese and Western medicine can significantly improve the efficacy of antibiotics, expand the antibacterial spectrum, give play to their respective advantages, and reduce side effects [26]. Traditional Chinese medicine preparations are widely used clinically with antibiotics including AZM. AZM is widely used in clinical practice together with traditional Chinese medicine preparations for clearing away heat and detoxification (such as Xiyanping injection, Tanreqing injection, etc.) and has a good clinical therapeutic effect [27, 28].

*In vitro* bacteriostatic experiments showed that combination medication showed synergistic/additive effects on standard strains *Staphylococcus aureus, Streptococcus pneumoniae, Hemolytic streptococcus, Shigella dysenteriae,* and *Klebsiella pneumonia*. The inhibitory effect of combination medication on drug-resistant strains was evaluated in *vitro*. The MIC value of the KM group on a series of highly resistant AZM did not change indicating that the induction of high resistance to AZM did not change the inhibitory effect of KM on corresponding strains. Based on the determination of the MIC values of five highly drug-resistant strains, the combination of the two drugs significantly reduced the MIC values of highly drug-resistant bacteria to 32 *μ*g/mL and 64 *μ*g/mL, which effectively improved AZM resistance. Preliminary studies show that the combination medication has an antibacterial effect on drug-resistant strains in *vitro*.

LPS-induced RAW264.7 cells were used to establish an inflammation model in *vitro*, and the inhibitory effects of azithromycin combined with KM on the inflammatory factors TNF-*α*, NO, and IL-6 were evaluated. Compared with the single drug group, the combined drug group has a significant inhibitory effect on the inflammatory factors TNF-*α*, NO, and IL-6. The result showed KM-AZM combination has a more significant anti-inflammatory effect; at the same time, the 1 : 1 dose relationship ratio group has the best anti-inflammatory effect. In the study, only three kinds of inflammatory indicators such as TNF-*α*, NO, and IL-6 were investigated, and inflammatory indicators such as NF-*κ*B and IL-8 need to be further studied in order to improve the reliability and systematicity of the study.

It is well known that the MAPK signaling pathway has a crucial effect on the body and is involved in a series of cellular responses triggered by environmental and developmental signal transduction, including cell survival, proliferation, differentiation, inflammation, and apoptosis. The three most common MAPKs in human cells are ERK, JNK, and p38 kinase. NF-*κ*B is an important transcription regulator in the human body, and it is a protein family composed of multiple polypeptide subunits. p65, I*κ*B, and p-I*κ*B are all important members of the NF-*κ*B family [29, 30]. In the current study, our data revealed that KM-AZM attenuated the LPS-induced lung histopathologic changes and inflammatory cytokine production and thus protected mice against LPS-induced induced acute lung injury. It is known that inflammatory stimuli such as LPS led to the activation of MAPK and the transcription factor NF-*κ*B, which mediates the expression of several proinflammatory cytokines, including TNF-*α* and IL-6, playing an important role in many inflammatory disease processes.

To extend that study, in the present research, we study the pharmacokinetics of AZM combined with KM and evaluate the metabolic process of the drug in rats. After KM-AZM combination administration, the average retention time of azithromycin increases, which enhances the retention time of the drug in the body and can exert a relatively good curative effect. At the same time, KM significantly increased the apparent volume of distribution of AZM after multidose combined administration. The distribution of the drug in the body after multiple administrations of the combination drug is more extensive.

The count of leukocytes and neutrophils in the whole blood of the model group was significantly higher than the blank group, and lung tissue pathological sections of the model group had a large number of inflammatory cell infiltration. In summary, the model group was successfully formed. The lung tissue homogenate count and lung tissue pathology results showed that the combination can significantly improve the treatment of pneumonia in rats and has a stronger anti-inflammatory effect than the single drug group. The inhibitory effect of azithromycin combined with Kumu injection on inflammatory factors TNF-*α* and IL-6 is not significantly different from that of the AZM single drug group. It is speculated that the combined use of the two drugs may have stronger inhibitory effects on other inflammatory factors, which needs to be determined by further study research. In *vitro* bacteriostatic experiments show that the combination of drugs has a good bacteriostatic effect on highly resistant *K. pneumoniae*, and the pharmacodynamic experiments of drug-resistant *K. pneumoniae* infected rats are worthy of further research.

## 5. Conclusions

In summary, AZM and KM in combination could inhibit the release of NO, TNF-*α*, and IL-6 *in vitro* and *in vivo*, probably by blocking the activation of NF-*κ*B and MAPKs signaling pathways. At the same time, KM-AZM can effectively alleviate the clinical symptoms of *K. pneumoniae* induced and improve the lung tissue lesions. These findings provide a new possibility for the clinical treatment of *K. pneumoniae* pneumonia and the basis for a wider range of clinical applications. Nevertheless, more studies are needed to decipher the underlying synergistic mechanism of KM-AZM.

## Figures and Tables

**Table 1:** MIC and FICI of AZM with KM against common bacterial strains.

Strain	Alone MIC (mg/L)		Com MIC (mg/L)		FICI	Outcome^∗^
	AZM	KM	AZM	KM		
*Standard strains*						
* S. aureus*	2	1	0.5	0.06	0.31	1
* E. coli*	8	0.5	2	0.03	0.31	1
* H. streptococcus*	2	0.25	1	0.06	0.74	2
* K. pneumoniae*	2	0.25	0.25	0.06	0.37	1
* S. pneumoniae*	1	0.5	0.25	0.12	0.49	1
* S. dysenteriae*	2	1	0.5	0.12	0.37	1
* P. aeruginosa*	4	2	4	2	2	3

*Resistant strains*						
* S. aureus*	256	1	32	0.25	0.38	1
* E. coli*	256	0.5	64	0.125	0.5	1
* K. pneumoniae*	256	0.25	32	0.06	0.37	1
* S. pneumoniae*	256	0.5	64	0.125	0.5	1
* S. dysenteriae*	256	1	64	0.125	0.8	1

Note: ^∗^1 stood for synergy with FICI ≤0.5, 2 for additive with FICI ranging between 0.5 and 1, and 3 for indifference FICI ranging between 1 and 2.

**Table 2:** The *Q* value for KM-AZM combination on *in vitro* release of inflammatory cytokines.

KM-AZM compatibility ratio		*Q* value		
		NO	TNF-*α*	IL-6
2 : 1	25 *μ*g/mL : 12.5 *μ*g/mL	0.87	1.24	1.25
	50 *μ*g/mL : 25 *μ*g/mL	0.93	1.22	1.42
	100 *μ*g/mL : 50 *μ*g/mL	0.90	1.16	1.33
	25 *μ*g/mL : 25 *μ*g/mL	0.90	1.39	1.18

1 : 1	50 *μ*g/mL : 50 *μ*g/mL	0.92	1.23	1.24
	100 *μ*g/mL : 100 *μ*g/mL	0.93	1.22	1.42
	25 *μ*g/mL : 50 *μ*g/mL	0.95	1.22	1.17

1 : 2	50 *μ*g/mL : 100 *μ*g/mL	0.93	1.17	1.22
	100 *μ*g/mL : 200 *μ*g/mL	0.97	1.19	1.36

**Table 3:** Pharmacokinetic parameters for AZM in rats *i.v.* administered multidose of AZM with or without an equivalent dose of KM (both 75.0 mg/kg once daily for 7 days).

Parameters	AZM alone	KM + AZM
AUC_0-*t*_ (*μ*g/L·h)	7813.66 ± 1120.12	6274.32 ± 3170.05
MRT_0-*t*_ (h)	4.93 ± 0.40	8.38 ± 0.32^*∗*^
*t*_1/2_ (h)	7.54 ± 2.76	9.12 ± 2.97
CL (L/h/kg)	8.80 ± 1.71	7.55 ± 5.29
*V*_*d*_ (L/kg)	92.83 ± 23.68	150.95 ± 34.64^*∗*^

^*∗*^ *P* < 0.05 indicated a significant difference from the AZM alone.

## Data Availability

The data used to support the findings of this study are included within the article.

## Abbreviations

AZM: Azithromycin
KM: Kumu injection
TCM: Traditional Chinese medicine
MIC: Minimum inhibitory concentration
FICI: Fractional inhibitory concentration index
CLSI: Clinical and Laboratory Standards Institute
CFU: Colony-forming unit
MRM: Multiple reaction monitoring
MRT: Mean residence time
AUC: Area under curve
*t*_1/2_: Half-life
*k*: Elimination rate constant
MRT: Mean residence time
TNF-*α*: Tumor necrosis factor-*α*
IL-6: Interleukin-6
NF-*κ*B: Nuclear factor-*κ*B
MAPK: Mitogen-activated protein kinase
I*κ*B: Inhibitor of *κ*B
JNK: Jun *N*-terminal kinase
ERK: Extracellular signal-regulated kinase.
